# Therapeutics of Infancy and Childhood

**Published:** 1898-06

**Authors:** 


THERAPEUTICS OF INFANCY AND CHILDHOOD. By A. JACOBI, M. D., Clinical Professor of the Diseases of Children in the College of Physicians and Surgeons, New York, etc. Crown octavo, pp. xvi.—629. Second edition. Philadelphia: J. B. Lippincott Company. 1898.

The first edition of this work was noticed so recently in the JOURNAL that an extended review is not necessary. The present edition has been thoroughly revised, giving the work a completeness which was lacking in the first. It was then one of the most excellent works of its kind ever issued, and the revision has only added to its excellence. The book is still a personal one, a fact which enhances its value in these days of unpersonal systems. M. J. F.

